# Molecular evidence of Zn chelation of the procaspase activating compound B-PAC-1 in B cell lymphoma

**DOI:** 10.18632/oncotarget.6505

**Published:** 2015-12-07

**Authors:** Aloke Sarkar, Kumudha Balakrishnan, Jefferson Chen, Viralkumar Patel, Sattva S. Neelapu, John S. McMurray, Varsha Gandhi

**Affiliations:** ^1^ Department of Experimental Therapeutics, The University of Texas Health Science Center, Houston, Texas, USA; ^2^ Department of Lymphoma and Myeloma, The University of Texas Health Science Center, Houston, Texas, USA; ^3^ Department of Leukemia, UT MD Anderson Cancer Center, Houston, Texas, USA; ^4^ Graduate School of Biomedical Sciences, The University of Texas Health Science Center, Houston, Texas, USA

**Keywords:** lymphoma, MCL, pro-caspase-3, Zn-ligands, B-PAC-1

## Abstract

The resistance of apoptosis in cancer cells is pivotal for their survival and is typically ruled by mutations or dysregulation of core apoptotic cascade. Mantle cell lymphoma (MCL) is a non-Hodgkin's B-cell malignancy expressing higher anti-apoptotic proteins providing survival advantage. B-PAC-1, a procaspase activating compound, induces apoptosis by sequestering Zn bound to procaspase-3, but the amino acids holding Zn in Caspase-3 is not known. Here we show that reintroduction of WT caspase-3 or 7 in Caspase3–7 double knock-out (DKO) mouse embryonic fibroblasts (MEF) promoted B-PAC-1 to induce apoptosis (27–43%), but not in DKO MEFs or MEFs expressing respective Casp3–7 catalytic mutants (12–13%). Using caspase-6 and -9 exosite analysis, we identified and mutated predicted Zn-ligands in caspase-3 (H108A, C148S and E272A) and overexpressed into DKO MEFs. Mutants carrying E272A abrogated Zn-reversal of apoptosis induced by B-PAC-1 via higher XIAP and smac expressions but not in H108A or C148S mutants. Co-immunoprecipitation analysis revealed stronger XIAP-caspase-3 interaction suggesting a novel mechanism of impulsive apoptosis resistance by disrupting predicted Zn-ligands in caspase-3. B-PAC-1 sponsored apoptosis in MCL cell lines (30–73%) via caspase-3 and PARP cleavages accompanied by loss of Mcl-1 and IAPs including XIAP while Zn substantially abrogated B-PAC-1-driven apoptosis (18–36%). In contrary, Zn is dispensable to inhibit staurosporin, bendamustine, ABT199 or MK206-induced apoptosis. Consistent to cell lines, B-PAC-1 stimulated cell death in primary B-lymphoma cells via caspase-3 cleavage with decline in both Mcl-1 and XIAP. This study underscores the first genetic evidence that B-PAC-1 driven apoptosis is mediated via Zn chelation.

## INTRODUCTION

Mantle cell lymphoma (MCL) is a fatal B-cell non-Hodgkin's malignancy with a tendency to disseminate throughout the body, including lymphoid tissues, bone marrow and peripheral blood there by offering a broad spectrum of clinical, pathological, and biological significance. Cyclin D1 over-expression owing to reciprocal translocation event t(11;14)(q13;q32) [1], accompanied by constitutively activated PI3K/AKT/mTOR network, aberrant WNT, Hedgehog and NF-κB pathways are the major forces that drive MCL cell proliferation and survival [2–5]. All these signaling events converge to resistance to apoptosis which in part caused by over-expression of anti-apoptotic Bcl-2 family members including Bcl-2 and Mcl-1 in majority of MCL cells. Given the potential role(s) of pro-apoptotic compounds for treating cancer, incessant efforts are in progress to develop therapeutics that target specific proteins in the apoptotic cascade. Activation of zymogen procaspase-3 (pro-casp3) to caspase-3 (Casp3) is the hallmark of cell-death pathway and the key “executioner” caspase, catalyzing the hydrolysis of a multitude of protein substrates within the cell [6]. Caspases play a provocative role leading to programmed cell death (PCD) and are classified as either initiators or upstream (Casp2, 8 and 9) or executioners or downstream (Casp3, 6 and 7). Executioner caspases are controlled via zymogen activation, in which proteolytic processing by an upstream protease results in the generation of a cleaved, active protein [7]. These caspase zymogens are inherently maintained in a full-length, unprocessed, and inactive dimer before being processed during the onset of PCD. Several targets of apoptotic machinery include genotoxic agents, MDM2-p53 or B-cell receptor pathway inhibitors and Bcl2 antagonists [8–11] rely on upstream apoptotic cascades yet a few are known to induce directly Casp3–7 bypassing defective upstream apoptotic circuitry. Interestingly, majority of cancer cells including MCL possess relatively higher levels of pro-casp3 [12] and pro-casp7 [13] allowing a rational approach to selectively induce PCD via direct activation of caspases. Therefore targeting cancer cells expressing higher level of pro-casp3 over normal cells would be advantageous. This strategy of direct caspase activation and exterminating upstream regulators of PCD that otherwise help MCL cells to acquire a strong survival advantage is becoming a powerful tool in recent years.

Procaspase-activating compound-1 (PAC-1) was discovered as the first line of procasp3–7 activators along with other PAC-1 derivatives [12, 14–16]. A fluorescent derivative of PAC-1 was found to co-localize within the apoptotic cells expressing Casp3–7 suggest that PAC-1 acts to chelate inhibitory Zn from procasp3–7 to induce PCD [12, 14–16]. Second generation of PAC-1 derivatives resulted in more potent analog such as B-PAC-1. We describe here the genetic basis of Zn chelation and caspase activation by B-PAC-1 that directly activate PCD in both MCL cell lines and in primary lymphoma cells with a promise for future therapeutic use.

## RESULTS

### B-PAC-1 induced cell death in MCL via Casp3 cleavage: reversal of apoptosis by Zn

**Figure 1:**
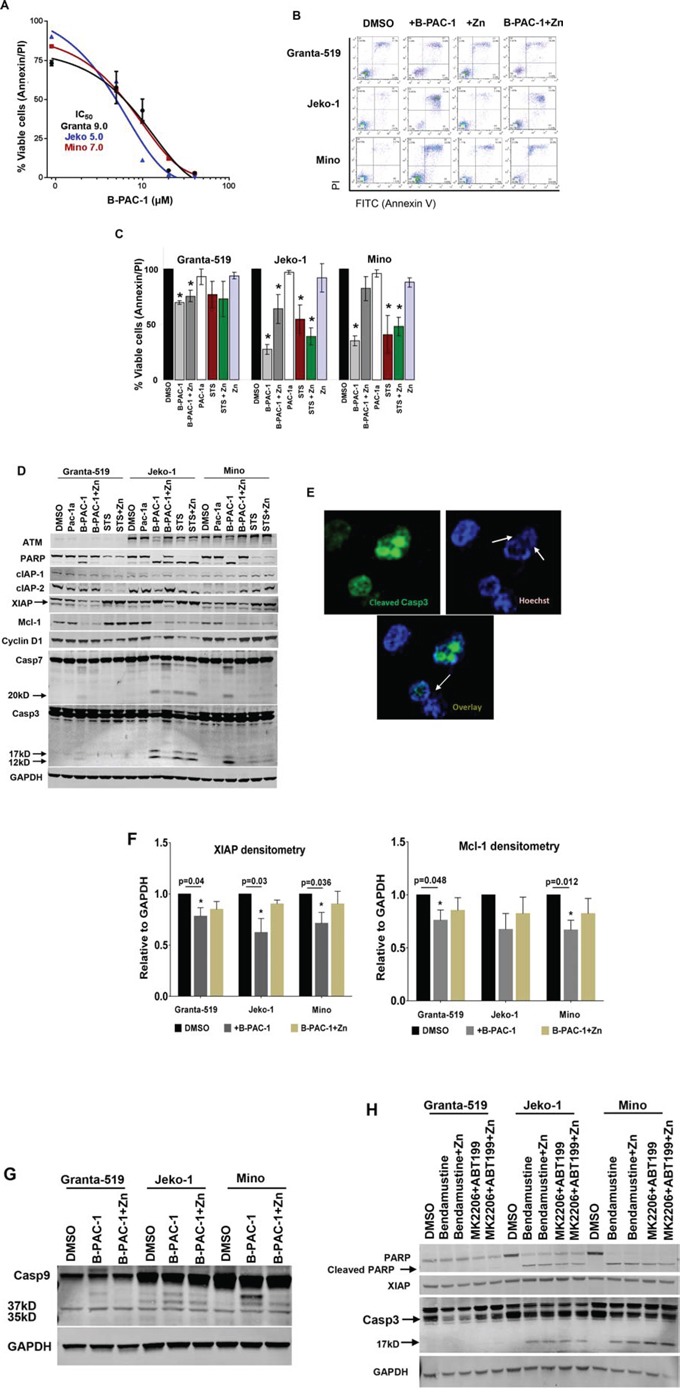
B-PAC-1 induce cell death in MCL cell lines by activating Casp3 **A.** Dose dependent cell death by B-PAC-1 in MCL cell lines. Granta-519, Jeko-1 and Mino cells were incubated with increasing concentration of B-PAC-1 for 24 hr, stained with Annexin V-PI and acquired in FACS caliber for cell death analysis. Their respective IC_50_ value was determined as shown in the inset. **B.** FACS analysis showing differential cell death induced by B-PAC-1 (10 μM, 24 hr) with or without Zn in Granta-519, Jeko-1 or Mino cells. **C.** Annexin V-PI FACS analysis of B-PAC-1 induced cell death (Granta-519; *p < 0.0001; Jeko-1; *p < 0.0001 and Mino; *p < 0.0001) or inhibition by Zn (Granta-519; *p < 0.007; Jeko-1; *p < 0.035) (*n* = 5; Mean ± SE) as described in B. Pac-1a, was used as negative control while staurosporine (STS;100 nM) was used as positive control (*n* = 3 *p < 0.03–0.004 in Granta-519; *p < 0.03–0.002 in Jeko-1 or *p < 0.020 - 0.003 in Mino cells compared with DMSO control. **D.** Western blot analysis of protein extracts (30 μg) from Granta-519, Jeko-1 and Mino cells treated with indicated compounds for 24 hr showing cleavage of Casp3 and Casp7 by B-PAC-1 and STS accompanied by cleavage of both Casp3 substrates ATM and PARP and corresponding loss of XIAP, Mcl-1, cIAP-1 and cIAP-2 proteins. Treatment with inactive Pac-1a (10 μM) was used as negative control and Zn was utilized to abrogate B-PAC-1 induced PCD. GAPDH was used for loading control. Identical blots were either reprobed or cut in strips and separately probed with antibodies for indicated proteins. **E.** Immunofluorescence analysis of Jeko-1 cells treated with B-PAC-1 for 24 hr showing Casp3 cleavage is accompanied by nucleosomal pyknosis. Arrows indicate nuclear pyknosis in cleaved Casp3 expressing cells. **F.** Densitometry analysis (*n* = 4; Mean ± SE) showing loss of anti-apoptotic proteins XIAP and Mcl-1 following treatment with B-PAC-1 and Zn in Granta-519, Jeko-1 and Mino cells. *Significant difference from control. **G.** Western blot analysis (30 μg) of protein extracts from Granta-519, Jeko-1 and Mino cells showing cleavage of Casp9. Arrows indicating 37 and 35kD cleaved bands. GAPDH was used for loading control. **H.** Western blot (30 μg) analysis showing cleavage of Casp3 and PARP and loss of XIAP in MCL cell lines treated with Bendamustine (30 μM) or a combination of ABT199 (20 μM) and MK2206 (5 μM) for 24 hr in presence or absence of Zn (100 nM). GAPDH was used for loading control.

MCL cell lines treated with B-PAC-1 for 24 hr in a dose-dependent manner and their respective IC50 values were determined as ∼9.0, ∼5.0 or ∼7.0 μM for Granta-519, Jeko-1 and Mino cells, respectively (Figure 1A). Based on this IC_50_ value, we selected 10 μM for all additional experiments unless specied otherwise. Co-incubation of B-PAC-1 with Zn rescued cell death in these cells entailing that Zn plays a role in B-PAC-1-induced PCD (Figure 1B). Treatment with either Zn or Pac-1a (an inactive derivative of B-PAC-1∼10 μM) failed to accomplish detectable PCD compared to DMSO control (Figure 1C). In contrast, staurosporin-induced apoptosis was not reverted by Zn (Figure 1C).

Western blot analysis from cells treated with either B-PAC-1 or STS revealed detectable cleavage of Casp3 substrate PARP (poly ADP ribose polymerase). Interestingly, both Annexin V-PI FACS analysis and protein analysis revealed that ATM deficient [19] Granta-519 was relatively resistant to B-PAC-1-induced PCD compared to ATM proficient Jeko-1 and Mino cells. In contrast, regardless of p53 status, both p53 deficient Jeko-1 and p53 proficient Mino cells [19] were equally sensitive to B-PAC-1 as evidenced by the cleavage of both executioner Casp3 (p17 and p12), Casp7 (p20) and PARP (Figure 1D). Immunoblot assays suggested that multiple anti-apoptotic proteins including IAPs (cIAP-1, cIAP-2 and XIAP), Mcl-1 and cyclin D1 levels were reduced following B-PAC-1 treatment. This observation was further supported by direct immunofluorescence analysis from Jeko-1 cells (Figure 1E) indicating B-PAC-1 induced Casp3 cleavage is accompanied by nuclear pycnosis and membrane blebbing. Densitometry analysis (Figure 1F) revealed a significant decline in both XIAP and Mcl-1 protein levels following B-PAC-1 treatment. Consistent with Annexin V-PI FACS data, co-incubation of B-PAC-1 and Zn also restored XIAP and Mcl-1 proteins, inhibition of Casp3 and Casp7 cleavage and their substrates including PARP and ATM (Figure 1D). Amongst other caspases, B-PAC-1-induced cleavage of initiator Casp9 was inhibited by Zn while Casp6 cleavage was not detected (data not shown) (Figure 1G). The DNA alkylating agent bendamustine, Bcl-2 antagonist ABT199 or pan-AKT inhibitor MK2206 are clinically used for treatment of B-cell malignancies. These agents also induced PCD; however co-incubation of these compounds with Zn failed to rescue apoptosis. This study indicates that Zn induced reversal of PCD is B-PAC-1-specific (Figure 1H).

### Casp3-7 DKO MEFs were resistant to B-PAC-1-driven apoptosis

**Figure 2:**
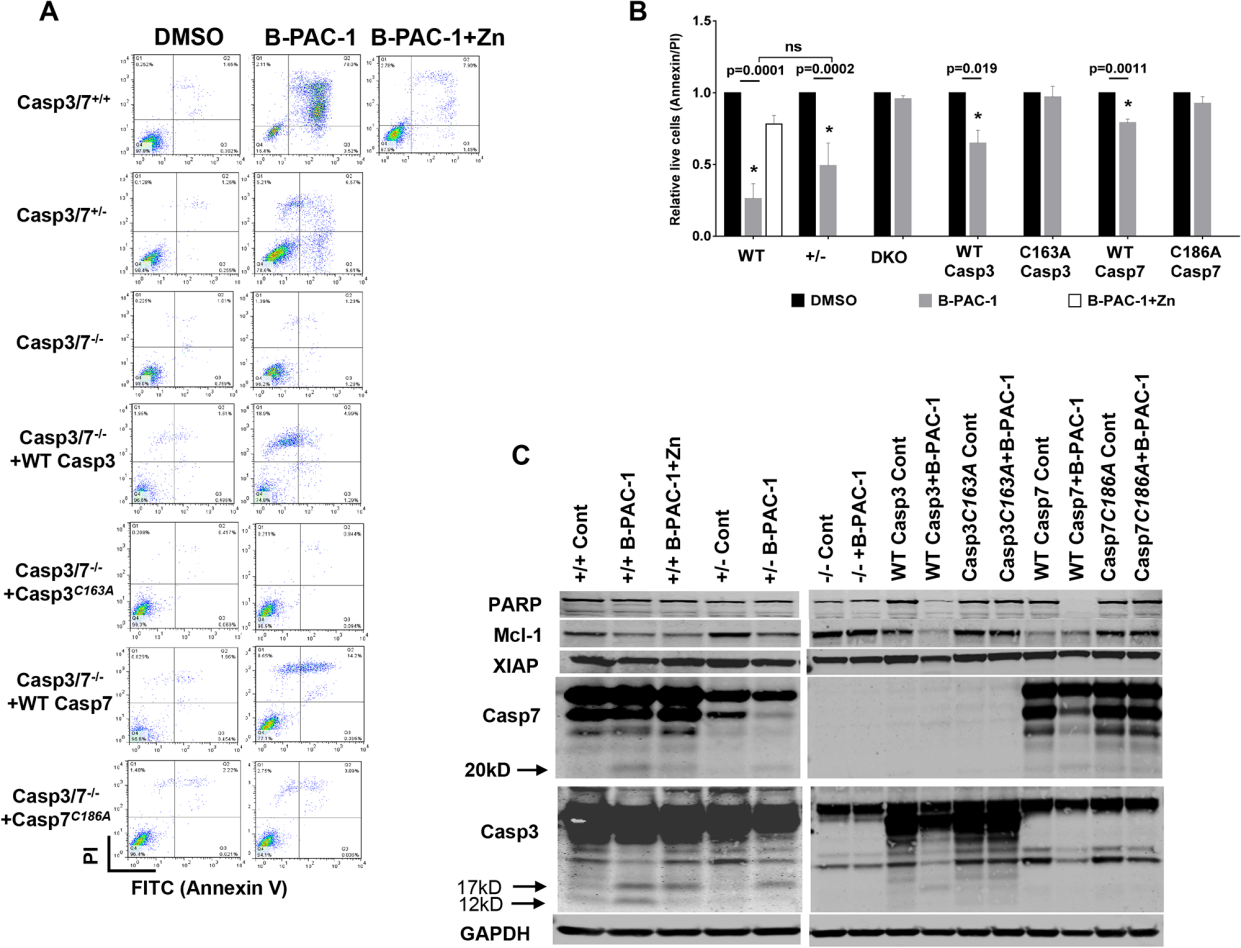
Casapse3/7 DKO MEF cells are resistant to B-PAC-1 induced cell death **A.** Annexin V-PI FACS analysis of B-PAC-1 induced cell death in the indicated MEF cells with either a WT Casp3 or Casp7 or MEF cells carrying a mutation in the respective catalytic sites. DMSO was used as control. **B.** Cells were treated in triplicate as in 2A and relative live cells (Annexin V-PI) were plotted as percent of control mean (*n* = 3 ± SE). *Significant difference from control. **C.** Immunoblot analysis of protein isolated from indicated cells before and after treatment with B-PAC-1 (10 μM for 24 hr). For PARP, XIAP and Mcl-1, 50 μg protein was used while 250 μg was loaded for Casp3 and Casp7. GAPDH (50 μg from same extracts) was used for loading control.

Further explication of the role of Casp3 and Casp7 during B-PAC-1-induced apoptosis was evaluated in a series of MEF cell lines. While B-PAC-1 (10 μM for 24 hr) sensitized WT MEF (Casp3-7+/+) to PCD, Zn (100 nM for 24 hr), mostly inhibited B-PAC-1 action. In contrast, MEF cells lacking a single allele for both Casp3 and -7 (Casp3-7+/−) were less sensitive to B-PAC-1-driven apoptosis compared with WT MEF. As expected B-PAC-1 failed to sensitize DKO MEF cells to induce PCD, suggesting dependence of Casp3 and -7 for final execution of PCD by B-PAC-1 (Figure 2A and 2B).

Stable reintroduction of WT Casp3 into DKO MEF cells significantly sensitized B-PAC-1 induced PCD, while Casp3 catalytic mutant (Casp3C163A) blunted the effect of B-PAC-1 suggesting that Casp3 with an active site cysteine in the nucleophilic attack site is required for targeting B-PAC-1 to induce PCD. Although less sensitive, B-PAC-1 still induced PCD in DKO MEF cells stably transfected with WT Casp7 but not in Casp7 catalytic mutant (Casp7C186A) reconfirming that both Casp3 and -7 are important watchdogs for the final execution of B-PAC-1-induced apoptosis (Figure 2A and 2B). Western blot analysis revealed B-PAC-1 elicit depletion of full length PARP, Mcl-1 and XIAP levels accompanied by cleavage of Casp3 (17 and 12 KD) or Casp7 (20kD) in both WT and in Casp3-7+/− MEF cells. AnnexinV-PI FACS analysis reconfirmed B-PAC-1 driven PCD into DKO MEF cells carrying WT Casp3, Casp7 or Casp3-7+/− but not in DKO MEF cells carrying respective catalytic mutant versions (Figure 2C).

### Identification of the predicted Zn-binding amino acids in Casp3

**Figure 3:**
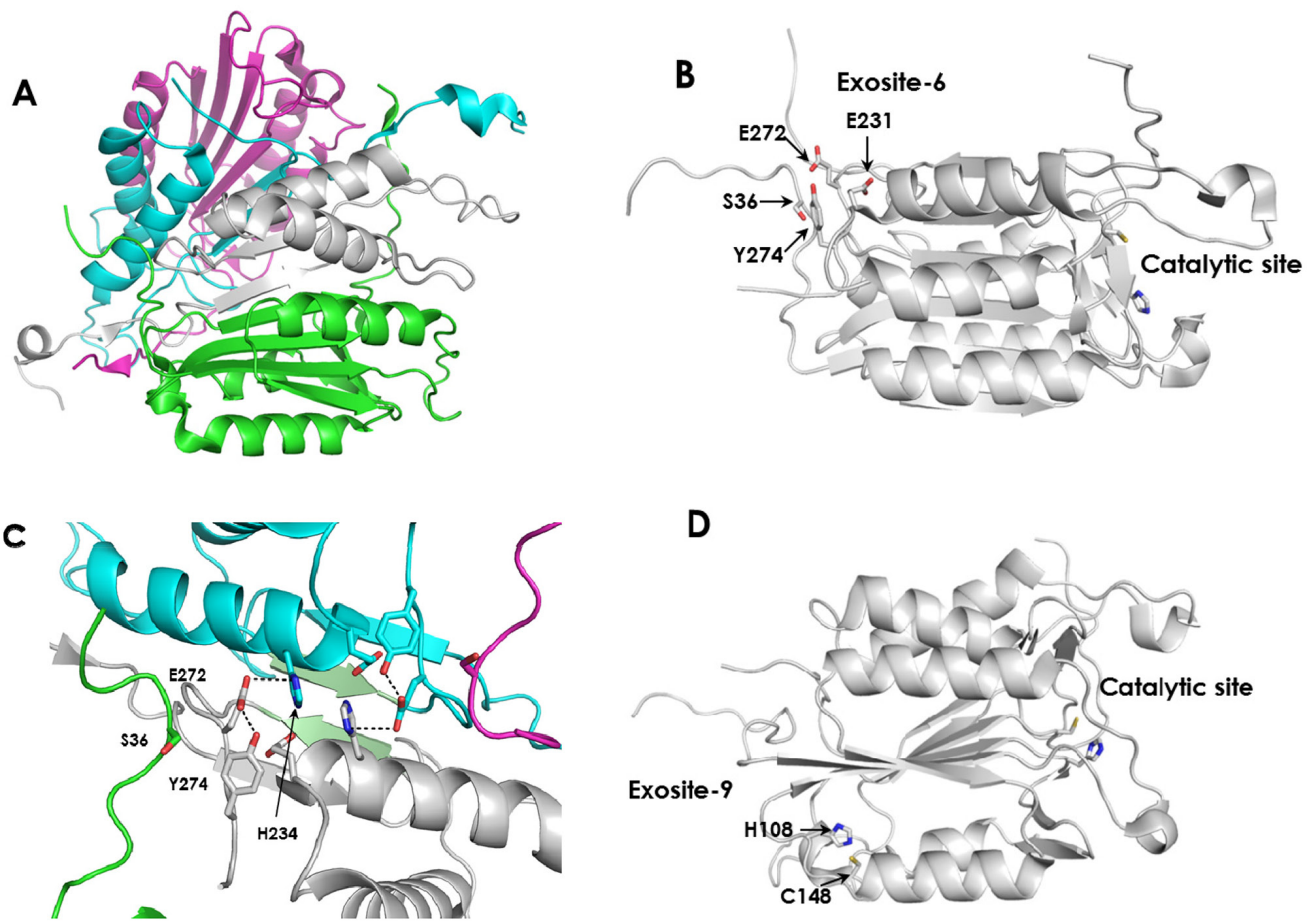
The structure of active Casp3 and the locations of potential Zn binding sites **A.** Casp3 as a dimer of heterodimers. The large and small subunits of one molecule of active Casp3 are colored green and white respectively. The corresponding subunits of the second Casp3 are colored magenta and cyan, respectively. (From PDBID 1I30). **B.** Exosite-6 is located distal from the catalytic site. **C.** Intra-subunit and inter-subunit interactions of E272 in exosite-6. The small subunit of the first Casp3 protein is colored white and the region of the large subunit containing Ser36 is colored green. The small subunit of the second Casp3 protein is colored cyan and the region of the large subunit containing Ser36 is colored magenta. Interacting β-sheets 264–267 from the opposing small subunits are colored light green. Hydrogen bonds between Glu272 and Tyr274 are depicted by black dashed lines. Potential inter-subunit hydrogen bonds between Glu272 and His234 are also indicated by black dashed lines. (from PDBID 1I30). **D.** Exosite-9 is located distal from the active site. Panels B and C were derived from PDBID 2J30.

Active Casp3 is a dimer of two cleaved procaspases as illustrated in Figure 3A and its catalytic activity is inhibited by Zn [20–21]. A recent report showed that three atoms of zinc bind to Casp3. Presuming that one Zn binds to the catalytic site, a common trait for all cysteine proteases, it was hypothesized that the other two zinc atoms may modulate catalytic activity, as reported for Casp6 and Casp9 [22]. The locations of the two non-catalytic zinc binding sites in Casp3 are currently unknown. Velaquez-Delgado and Hardy [23] recently reported that Casp6 binds two Zn atoms and using X-ray crystallography studies determined the binding site of the second metal ion, the Casp6 exosite. Occupation of this site by Zn has an inhibitory effect on the catalytic activity. Casp9 binds two Zn ions and site directed mutagenesis, determined the location of the second ion, the Casp9 exosite [22]. Using sequence and structural alignment, Velaquez-Delgado and Hardy [23] reported that in Casp3, the Casp6 exosite, (thereafter referred to as exosite-6), is comprised of Ser36, Glu272, and Tyr274 (Figure 3B). As in Casp6, with respect to the location of the catalytic site, these amino acids reside at the distal end of an α-helix, as shown in Casp3 crystal structure (PDBID 1PAU) [24–26]. This exosite does not contain the canonical zinc binding residues cysteine and histidine, but serine, glutamic acid, and tyrosine, and nearby Glu231 could potentially form a unique chelation site. Further, these residues could participate in intra- and inter-subunit interactions that could impact activity (Figure 3C). In Casp3, the Casp9 exosite, (exosite-9), is composed of His108, Ser113, Phe114, and Cys148. Of these, the X-ray structures of Casp3 show that the histidine and cysteine residues are positioned to chelate zinc and this site is also located distal from the catalytic site (Figure 3D). This site is highly conserved in all caspases [23]. To determine if either of these potential exosites has a bearing on B-PAC-1-driven activity of Casp3, mutations of key residues were introduced. In exosite-6, Glu272 was mutated to alanine. In exosite-9, His108 and Cys148 were mutated to alanine and serine respectively.

**Figure 4:**
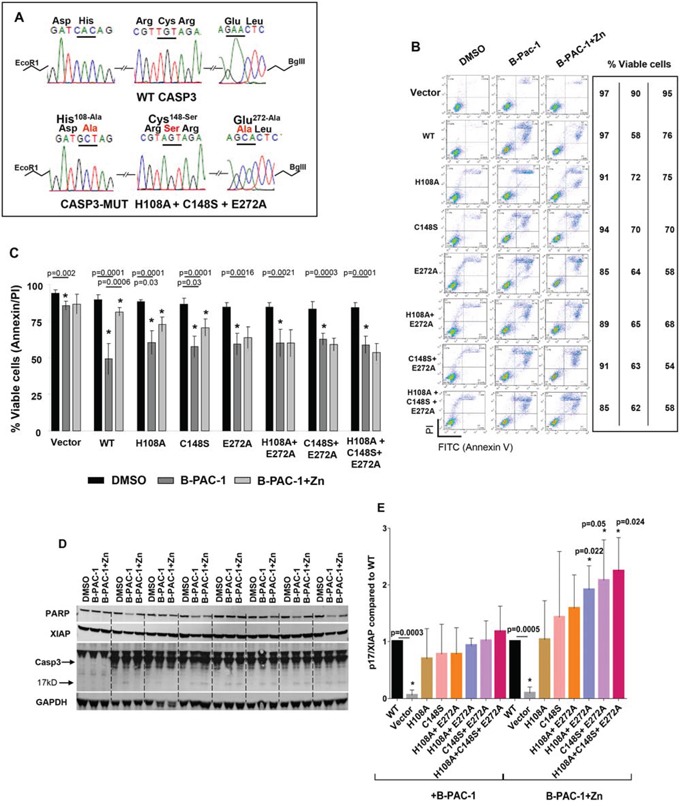
Mutation of predicted Zn binding regions in Casp3 preprotein abolishes Zn reversal of apoptosis induced by B-PAC-1 in MEF cells **A.** Nucleotide and amino acid sequence analysis showing WT and mutated Zn binding regions of Casp3 protein. **B.** Annexin V-PI FACS analysis showing cell death induced by B-PAC-1 (15 μM for 24 hr) with or without Zn (90 μM for 24 hr) in vector control Casp3–7 DKO, WT Casp3 or in MEFs stably transected with Casp3 constructs harboring mutated Zn binding amino acids. **C.** MEFs were treated as in B and mean (*n* = 5; ± SE) values relative to DMSO control are plotted. *Significant difference from control. **D.** Immunoblot analysis of total protein isolated from indicated cells showing cleavage of Casp3; (arrow indicates cleaved Casp3 band), XIAP, and full length PARP. GAPDH (250 μg from similar extracts) was probed for loading control. **E.** Experiment was conducted as in D showing mean (*n* = 3 ± SEM) densitometry analysis of cleaved Casp3 (p17) and XIAP ratios compared to WT Casp3 transfected MEF cells. *Significant difference from control.

A series of Flag-tagged Casp3 constructs harboring mutations were generated into the human Casp3 ORF and sequence verified as shown in Figure 4A. All these constructs were sub-cloned into a C terminal 3XFlag-epitope tagged vector and were stably transected into DKO MEF cells. Both B-PAC-1 and Zn IC_50_ values were titrated using WT Casp3 transfected DKO MEF cells (Supplemental information S1 and S2) and used for the entire experiment. While DKO MEF cells carrying vector alone failed to induce PCD, (Figure 4B), WT Casp3 transfected cells were sensitized by B-PAC-1 induced PCD and Zn significantly reverted apoptosis. Interestingly, cells carrying Casp3 mutants encountered a relative resistance to B-PAC-1-induced PCD compared with WT Casp3. B-PAC-1 induced PCD was significantly reversed by Zn in both H108A and C148S single mutants but failed to rescue in E272A single mutant or in double and triple mutants harboring E272A when compared with WT Casp3 (Figure 4C). Western blot analysis revealed B-PAC-1 driven PCD was accompanied by loss of full length PARP and XIAP and Casp3 cleavage (p17) in WT and Zn-Casp3 mutants (Figure 4D). Surprisingly, majority of Zn-Casp3 mutants expressed a relatively higher basal XIAP in presence of B-PAC-1 or B-PAC-1+Zn when normalized with cleaved Casp3 (p17) (Figure 4E). Neither basal pro-casp3 nor B-PAC-1 induced cleaved Casp3 (p17) fragment were altered in these clones. This data advocates a possible mechanism of resistance to spontaneous cleavage of apoptotic protease in these mutants via up-regulation of XIAP protein.

### Elevated Casp3-XIAP physical interaction and higher smac expression: inhibition of spontaneous apoptosis in DKO MEF cells carrying mutant Casp3

**Figure 5:**
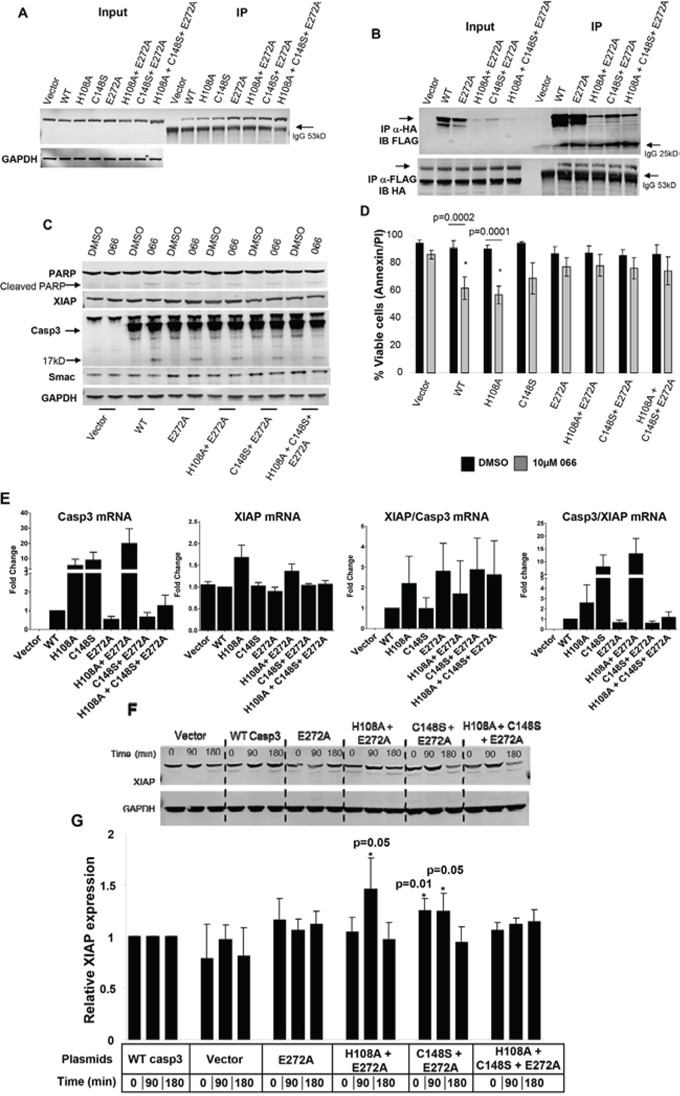
Enhanced Casp3-XIAP physical interaction in Casp3-Zn mutants **A.** Immunoprecipitation analysis (3000 μg) with anti-FLAG antibody showing endogenous XIAP levels in MEF cells engineered with WT and Casp3 mutants. 50 μg total extract was loaded as input control (left panel). Arrow indicates IgG heavy chain. **B.** Co-immunoprecipitation analysis using HEK293 cells transiently transfected with HA-XIAP and indicated Casp3 plasmids. 50 μg total extract was loaded as input control (left panel). Immunoprecipitated proteins were either probed with anti-FLAG antibody (upper panel; showing amount of Casp3 bound to XIAP) or anti-HA antibody showing amount of XIAP bound to Casp3 (lower panel). Arrow indicates IgG light (∼ 25kD upper panel) or heavy (∼53kD lower panel) chains. **C.** Immunoblot analysis of total protein isolated from stably transfected MEF cells with indicated plasmids showing smac mimetic (066) induced cleavage of Casp3 (250 μg; arrow indicating 17kD cleaved Casp3 band) and loss of full length PARP (250 μg), XIAP and smac (50 μg) protein expressions. GAPDH (50 μg from similar extracts) was probed for loading control. **D.** Annexin V-PI FACS analysis induced by smac mimetic (066) (*n* = 5; Mean ± SE) showing relative live cell in the indicated cell clones compared with individual DMSO controls. *Significant difference from control. **E.** Casp3 and XIAP mRNA expressions and their relative ratios in stably transfected MEF cells expressing vector, WT or Zn-Casp3 mutants. Cells were treated and processed for real-time RT-PCR assays. Data are mean ± SE (*n* = 3). **F.** Time course of cycloheximide (CHX) treatment in MEF cells showing XIAP protein expression (upper panel) in control, WT and Zn-Casp3 mutants. GAPDH was probed for loading control. **G.** Densitometry analysis (*n* = 5; Mean ± SE) showing relative XIAP protein levels in vector control, single (E272A), double (H108A+E272A and C148S+E271A) and triple (H108A+C148S+E272A) mutants compared with WT Casp3 transfected cells at identical time points. *Significant difference from control.

Having observed a relatively higher basal XIAP expression in majority of Zn-Casp3 mutants, we next tested Casp3-XIAP physical interaction in the mutant clones. Based on exosite cluster analysis, we hypothesized that by dismantling Zn ligands in Casp3 would spontaneously induce PCD. However, we could not detect any spontaneous PCD in these mutant clones. Immunoprecipitation analysis revealed a relatively higher Casp3-XIAP physical interaction in MEF cells carrying E272A mutation compared with WT Casp3 (Figure 5A). Alternatively, transient transfection assay into HEK293 cells co-transfected with vector, Casp3 (WT and all E272A harboring mutants) and HA-XIAP constructs demonstrated that an increased Casp3-XIAP physical interaction in majority of E272A harboring mutants compared with WT Casp3 transfected cells (Figure 5B). These data substantiate that mutation in predicted Zn-Casp3 amino acids reciprocally inhibit spontaneous PCD via acquiring an increased affinity of XIAP-Casp3 physical interaction.

Owing to stronger Casp3-XIAP complex in these mutants, we next hypothesized that perhaps more Smac mimetic is required in order to dismantle Casp3-XIAP interaction in order to induce PCD. MEF cells carrying WT Casp3 were titrated to determine IC_50_ value of 066 (Supplemental information S3). MEF cells carrying WT or Zn-Casp3 mutants were identically treated with 066 and their viability was determined by AnnexinV-PI FACS analysis (SI Information S4). Western blot analysis revealed 066-induced PCD was more pronounced in WT Casp3 carrying MEF cells accompanied by cleavage of PARP, loss of XIAP and smac levels and Casp3 (p17) cleavage compared to Zn-Casp3 mutants (Figure 5C and SI Information S5) while majority of these mutants retained relatively higher smac expression in identical condition. AnnexinV-PI FACS analysis reconfirmed that 066 significantly induced PCD (*p* < 0.0001–0.0002) in both WT and H108A mutant, while C148S and mutants carrying E272A were resistant to 066. This data prompted us to study real time RT-PCR analysis of Casp3 and XIAP mRNA levels in these clones. A relatively higher Casp3 mRNA expression was observed in H108A, C148S and in C148S+E272A double mutant compared with WT (Figure 5E and Supplemental information S5). In contrast, XIAP expressions remained unchanged in majority of clones except in H108A and C148S+E272A double mutants. However, the ratio of XIAP/Casp3 remained higher in these mutant clones compared with MEFs carrying WT Casp3. This observation supports protein expressions in transient transfection assay with either higher XIAP or lower Casp3 expressions leading to an increased affinity of XIAP-Casp3 physical interaction (Figure 5B). Although none of these mRNA expression data were significant, we predicted a possible stability of XIAP protein in these clones. Cycloheximide treatment in these clones followed by protein and densitometry analysis revealed a relatively higher XIAP stability in the double mutants harboring E272A compared with WT Casp3 (Figure 5F). Collectively, these studies demand a possible novel mechanism of resistance in these clones averting spontaneous PCD following mutation in the predicted Zn ligands in Casp3 via higher XIAP-Casp3 physical interaction, higher XIAP and smac expressions and XIAP stability.

### B-PAC-1 induces apoptosis in primary B-cell lymphoma cells

**Figure 6:**
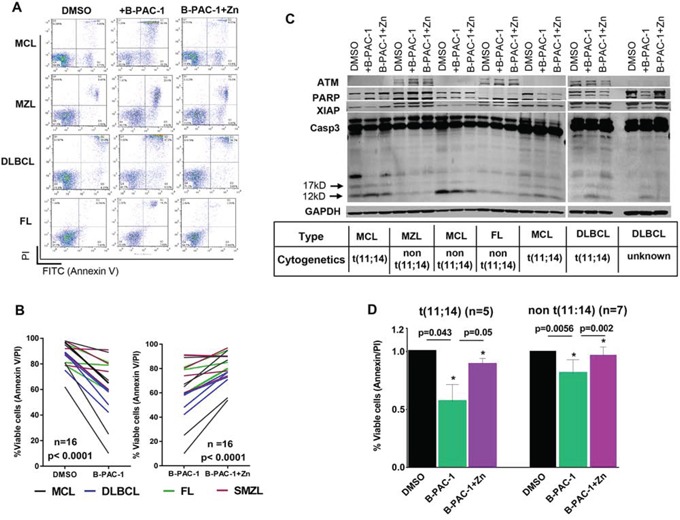
Primary lymphoma cells from patients are sensitive to B-PAC-1 induced cell death **A.** Annexin V-PI FACS analysis showing multiple B-cell primary lymphoma samples treated with B-PAC-1 (10 μM for 24 hr) with or without concomitant Zn incubation. **B.** B-PAC-1-mediated cell death and reversal of apoptosis by Zn in primary lymphomas were plotted (*n* = 16; *p* < 0.0001). **C.** Immunoblot analysis (30 μg) of a panel of primary lymphoma samples showing B-PAC-1 induced cell death or Zn inhibition of apoptosis as shown by Casp3 cleavage (p12 and p17 bands). Membrane was cut and fragments were probed with ATM, PARP and XIAP. GAPDH was used as loading control. **D.** Cell death induction by B-PAC-1 or reversal of apoptosis by Zn in MCL primary samples harboring t(11:14) (*n* = 5) and in non t(11;14) cytogenetics (*n* = 7). DMSO treatment was used as control. *Significant difference from control.

Primary lymphoma cells from 19 patients including MCL (*n* = 7), MZL (*n* = 5), DLBCL (*n* = 4) and FL (*n* = 3) were tested (Table 1). B-PAC-1-induced PCD was a common feature in all these samples regardless of their subtype while co-incubation with Zn resulted in significant inhibition of PCD (Figure 6A and 6B). Amongst different lymphomas (Figure 6B) MCL and DLBCL subtypes were more sensitive than MZL and FL samples. Western blot analysis showed B-PAC-1-induced cleavage of Casp3 and PARP together amid loss of XIAP (Figure 6C). In several primary cells, ATM protein expression was undetectable; a common feature in MCL [27]. Collectively, B-PAC-1 stimulates PCD in primary lymphomas regardless of ATM status (Figure 6D) and this was partially reverted by Zn addition.

**Table 1:** Patient characteristics and B-PAC-1 induced cell death

							% viable cells		
Pt #	Type of Disease	Age (yrs)	Gender	Cytogenetics	Cytogenetics	Additional information	DMSO	B-PAC-1	B-PAC-1 + Zn
1	MCL	60	F	t(11;14)	Complex	At initial diagnosis	97	65	95
2	MCL	60	F	t(11;14)	Complex	At initial diagnosis	95	67	87
3	MCL	43	M	t(11;14)	Complex	Refractory	62	10	54
4	MCL*	60	M	t(11;14)	6q-, +11q22.3 (ATM x3), 13q-	5 months after initial Dx	38	12	34
5	MCL	Unkn	Unkn	Unkn	Unknown	Unknown	98	89	90
6	MCL	57	M	t(11;14)	Diploid male karyotype 46,XY	At initial diagnosis	81	25	56
7	MCL*	61	F	t(11;14)	Complex	Relapsed and Refractory	53	33	42
8	SMZL	73	F	t(11;14)	Complex	Refractory	92	91	90
9	SMZL	75	M	Normal	Pseudodiploid clone 46,XY,del(7)(q22q34) [15]	Dx in 2008; on observation	88	60	74
10	MZL	39	M	Normal	Diploid male karyotype 46,XY [20]	Dx in 2010; on observation	98	80	97
11	MZL	74	M	Normal	Hyperdiploid male 47,XY,+Y [1]	At initial diagnosis	79	74	78
12	MZL*	71	F	Normal	Complex	Refractory	5	15	22
13	DLBCL	59	M	Normal	t(14;18)	Refractory	87	58	77
14	DLBCL	47	F	Unkn	Unknown	Refractory	89	48	77
15	DLBCL	72	F	Unkn	Unknown	Relapsed and Refractory	75	42	71
16	DLBCL	67	M	Unkn	Diploid with random chromosome loss 40∼43,XY[cp4]	At initial diagnosis	89	59	73
17	FL	57	F	Normal	Diploid female karyotype 46,XX [20]	Relapsed and Refractory	81	79	85
18	FL	57	F	Normal	Diploid female karyotype 46,XX [20]	Relapsed and Refractory	96	81	95
19	FL	68	M	Normal	Diploid male karyotype 46,XY [20]	At initial diagnosis	79	59	80

* Due to low viability of cells at collection, these samples were tested with B-PAC-1 and Zn but not included in analysis; Unkn, unknown;

## DISCUSSION

The treatment of MCL is an enormous challenge and is characterized by an aggressive clinical course and inevitable development of relapsed/refractory disease with a median overall survival time of only 3–5 years [28–30]. While immunotherapy with rituximab in combination with high-dose chemotherapy followed by autologous stem cell transplantation is a routine choice [31] yet, majority of patients experience poor outcome. High degree of genomic instability [32], distinct chromosomal alterations including losses, gains, and amplifications of chromosomal regions harboring gene(s) involved in cell-cycle regulation, DNA damage response pathways, signal transduction, and apoptosis are the major driving forces of MCL [1].

One of the stumbling blocks of treating MCL is accompanied by the deregulation of Bcl-2, Mcl-1 and BIM or inactivation of Apaf-1, thereby allowing these cells to evade caspase-mediated apoptosis [33, 34]. Higher expression of Bcl-2 family antiapoptotic proteins assisted by cyclin D1 mis-expression, faulty ubiquitin-proteasome system and defects in apoptosis upstream of caspase activation, renders MCL more difficult to treat [35]. Therefore, exploiting small-molecule that can directly activate pro-casp3 obliterating upstream regulators would be a more rational approach for targeting MCL [36, 37].

Earlier observation on pet dog bearing lymphoma [15] documented that S-PAC-1 activated procaspase-3 and elicit tumor cell death (an earlier derivative of B-PAC-1). In this study authors performed a small efficacy trial with S-PAC-1, which included six pet dogs bearing lymphoma and showed that S-PAC-1 was well tolerated and that the treatments induced partial tumor regression in four of six subjects. Furthermore a recent study documented with a detailed antitumor activity of PAC-1 (an earlier derivative of B-PAC-1) in murine xenograft model [38] carrying EL4 lymphoma cells transplanted in C57BL/6 mice and were treated with PAC-1 (125 mg/kg), 1541B (17.5 mg/kg), or with a combination of PAC-1 and 1541B (125 mg/kg and 17.5 mg/kg, respectively) once-a-day for three days. While PAC-1 had a small but statistically significant effect on tumor growth in this model, the combination of PAC-1 and 1541B dramatically retarded tumor growth *in vivo* without any systemic toxic side effects.

PAC-1 was synthesized to activate Casp3 by chelating inhibitory zinc ions from the zymogen pro-Casp3 initiating tumor cell death both *in vitro* and *in vivo* [14] and currently in Phase I clinical trial. B-PAC-1 was created [15] as an analogue of the first generation compound PAC-1. In this work, we test the efficacy of B-PAC-1 in MCL and present evidence showing B-PAC-1 has the ability to directly activate pro-casp3 to induce PCD in MCL cell lines via Zn sequestration and incubation of B-PAC-1 and Zn together significantly disrupt apoptotic program. We further show evidence that B-PAC-1 induces PCD in multiple primary lymphomas regardless of their cytogenetic status or their subtypes. Despite a variable magnitude of cell death offered by B-PAC-1 in the patient samples, consistent to our cell line studies we show that reversal of apoptosis via Zn chelation is widespread in primary lymphoma cells. However, normal T and B lymphocytes were less sensitive to B-PAC-1 mediated PCD providing a therapeutic index [39].

Despite a strong metal binding affinity exists in B-PAC-1 (containing the *ortho*-hydroxy N-acyl hydrozone motif), however, the Zn chelation is not widespread with other apoptosis-inducing drugs (Figure 1H). B-PAC-1 mediated Zn chelation was specific to executioner pro-caspases since B-PAC-1 does not alter the Zn-binding enzymes such as carboxypeptidase A or histone deacetylase [39]. Similarly, B-PAC-1 treatment did not influence inflammatory caspases like Casp1 [39] suggesting selectivity of B-PAC-1 for apoptotic terminal caspases.

Following a death stimuli, both intrinsic and the extrinsic apoptotic pathways converge and activate the “executioner” procaspases (pro-casp3, 7 and 6) through a cascade of events [40] leading to proteolysis of > 400 cellular proteins. We therefore assessed the role of B-PAC-1 on executioner caspases mostly in their respective nucleophilic sites. While initiator caspase-8 and -9 normally exist as inactive monomers that are activated by dimerization and not by cleavage [6] the effecter Casp3, 6 and 7 exist as dimers and are cleaved by initiator caspases leading to a conformational change that brings the two active sites together and creates a functional mature protease [41]. These procedural differences in the activation of initiator versus executioner caspases may result in selective action of B-PAC-1 on executioner caspases via Zn sequestration. Normally, Casp3 is a heterotetramer in its functional form and each heterodimer is formed and stabilized by hydrophobic interactions between the large (p17) and small (p12) subunits. The active site of Casp3 utilizes a cysteine-histidine dyad, with an aspartate in the P1 pocket. The active pocket of Casp3 is the nucleophilic cysteine163 and histidine121 [42]. Using DKO MEFs stably overexpressing Casp3-C163A or Casp7-C186A we show that B-PAC-1 failed to induce PCD reconfirming B-PAC-1 driven apoptosis requires catalytic cysteine moieties of both Casp3 and -7 regardless of Zn chelation for a full grown PCD.

Recently we documented B-PAC1 efficacy to induce apoptosis in both CLL and multiple myeloma cells [39, 43], however the mechanism of Zn chelation is not known. We therefore interrogated where Zn is bound to procaspase3 and how next generation PAC-1 compounds could be made more specific to caspase activation via Zn chelation. Historically, the role of Zn on apoptosis emerged from the pioneering study showing drastic PCD in the small intestinal crypts of zinc-deficient rats [44] or in leukemic cell lines [45] likely due to lack of zinc-mediated caspase inhibition. Essentially, glucocorticoid-induced PCD in mouse thymocytes was inhibited by Zn ions [46]. Although high zinc concentrations (>500 μM) induce PCD but at physiological level zinc is known as suppressor of apoptosis [47]. The inhibition of caspases by Zn and other transition metals, including copper, was reported soon after the discovery of caspases [21]. Zn preferentially binds to Cys (5.8%), His (2.4%), Asp (0.28%), and Glu (0.13%) (CHDE), collectively account for ∼98% of all Zn-binding residues [48]. Although majority (>90%) of cellular zinc is known to be sequestered in tightly-bound complexes of metalloenzymes and zinc finger proteins, however a small portion (<10%) of zinc exists in labile, loosely-bound pools [49]. Amongst other executioner caspases, Zn binds to Lys-36, Glu-244 and His-287 with a water molecule serving as the fourth ligand in Casp6 [23]. Similarly, H237, C239, and C287 include the active site dyad and are primarily responsible for zinc-mediated inhibition in Casp9 [22].

While the active sites for Zn in Casp3 molecule is unknown, we used the exosite cluster analysis of Casp9 [22] and mutated several predicted Zn-binding regions in both active and exosites. Our biochemical analysis shows that only E272 had an impact on apoptosis; while both H108 and C148 are not relevant sites since Zn could still significantly inhibit B-PAC-1-induced PCD in these mutants. Interestingly none of these clones were found to induce spontaneous apoptosis when over-expressed into DKO-MEF cells. In contrast, we encountered a unique mechanism of resistance to PCD from these mutant clones owing to relatively higher expression of XIAP and stronger Casp3-XIAP physical interaction rendering resistance to spontaneous apoptosis in these clones.

E272 is involved in several intra- and inter-domain interactions that may account for the observed effect (Figure 3C). Crystal structures of active Casp3 proposed multiple backbone-backbone interactions between the β-strands of the small subunits from residues 264–267 (Green β-strands in Figure 3C). Further, there are inter-domain hydrogen bonds between the side chains of both E272 and E231 of one small subunit and H234 of the opposing small subunit. Within each small subunit, the side chain carboxyl group of E272 contacts the side chain hydroxyl group of Y274, and both residues contact β and γ methylene groups of E231, the initial amino acid in α-helix 231–247. Additionally, the non-polar side chain atoms of E272 and Y274 in the small subunit contact the βCH_2_ of S36 in the large subunit. The carboxyl group of E272 is located within hydrogen bonding distance to H234 in the opposing small subunit in the dimeric unit. This network of interactions likely plays a role in stabilization of helix 231–247 and may be important for interaction with N-terminal segment of the large subunit. Replacement of E272 with alanine would remove the hydrogen bond with Y274 which could destabilize this cluster and alter the interactions between the large and small subunits of heterodimer. Further, disruption of this cluster could interrupt the interactions between the two small subunits thereby destabilizing the tetrameric structure of the enzyme complex which could alter the placement of the loop bundle [26] leading to impaired catalytic activity or altered substrate recognition. Further, the E272A mutation could alter the dynamics of helix 231–247 which could potentially impact catalytic activity in a manner analogous to the removal of zinc from the exosite in Casp6.

IAPs are unique molecules with BIR (baculovirus IAP repeat) domain and XIAP is the prototype member of the family. The complex structure of the Bir2 domain of XIAP and the catalytic domains of Casp3 indicates that the interaction takes place at the catalytic site and impact the loop bundle [25]. Given that the mutations in the exosites were rather distal from the catalytic site and from the sites of interaction of the Bir2 domain, it is not clear why the mutants would exhibit apparent increased affinity for XIAP. The mechanism by which these Casp3-Zn mutants inhibit spontaneous apoptosis awaits further studies although earlier predictions have suggested that H237 and C285 are involved in catalysis [50], yet no work has been reported using these targets and to our knowledge this study is the first documentation of how Casp3-Zn interaction is destabilized following mutation in the predicted exosites of Casp6 and Casp9.

## MATERIALS AND METHODS

### Cell lines and culture

Three MCL cell lines (Granta-519, JeKo-1 and Mino) were used in this study. Granta-519 was maintained in Dulbecco modified Eagle medium with high glucose (DMEM, Cellgro) supplemented with 20% FBS while both Jeko-1 and Mino cell lines were cultured in RPMI 1640 medium (ATCC) supplemented with 20% FBS. Wild type, heterozygote and double knockout mouse embryonic fibroblasts for Casp3 and Casp7 (DKO MEF) were maintained through passage 10 in DMEM (Cellgro) supplemented with 10% FBS. All media were supplemented with 1% penicillin-streptomycin (Invitrogen Inc.). These cell lines were routinely tested for *Mycoplasma* using a MycoTect kit (Invitrogen). MCL cell lines were validated by AmpF/STR Identification kit (Applied Biosystems) in the MD Anderson cell line validation core facility. Routine cell number and the mean cell volume were determined by Coulter channelyzer (Coulter Electronics).

### Patients and primary cell culture

For primary lymphomas, informed consent from every patient was obtained before inclusion in the study and was in accordance to the declaration of Helsinki. All protocols were approved by the Institutional Review Board at MD Anderson Cancer Center. Malignant lymphocytes from peripheral blood or from apheresis samples were isolated by Ficoll-Hypaque as described [17]. Cells were incubated in RPMI 1640 medium (ATCC) supplemented with 20% FBS and 1% penicillin-streptomycin.

### Reagents and antibodies

B-PAC-1 and PAC-1a, were > 98% pure and were provided by Prof. PJ Hergenrother, University of Illinois [14, 16]. Smac 066 was kindly provided by Dr. P. Seneci, University of Milan. All reagents and antibodies are listed in Supplementary Table 1S.

### Cloning and constructs

Plasmids 11813 (pcDNA3-Casp3-myc); 11814 (pcDNA3-Casp3 C163A-myc); 11815 (pcDNA3-Casp7-Flag); 11816 (pcDNA3-Casp7 C186A-Flag) and HA-XIAP (plasmid 25674: pEBB-HA-hILP) were obtained from Addgene. For Casp3–Zn mutation plasmids, a WT Casp3 construct was generated by cloning a PCR-amplified full-length human Casp3 coding region into EcoR1-BglII sites of p3XCMV-14 (Sigma) vector upstream of C terminal Flag epitope. Mutations in predicted Casp3-Zn binding amino acids were made by two-step PCR-based strategy. The following primers were used: 5′-GAAGATGCTAGCAAAAGGAGCAG-3′ for generating Casp3 H108-A mutation; 5′-TTTTTCAGAGGGGATCGTAGTAGAAG-3′ for generating Casp3 C148S mutation while Casp3 E272A mutation was introduced into a common reverse complement that contained a BglII cloning site (5′-CGATATCAGATCTATTTAGTGATAAAAAT AGAGTGCTTTTGTGA-3′). Mutated nucleotides are underlined. Similar two step PCR strategy was employed to create double (H108A and C148S or C148S and E272A) or triple (H108A, C148S and E272A) mutants. All plasmids were sequence verified at MD Anderson DNA sequencing core facility and cloned into EcoR1-BglII sites of p3XCMV-14 (Sigma).

### Transfection

DKO MEF cells were transfected overnight with 3 μg of p3X-CMV FLAG-Casp3 WT or Casp3-Zn mutant plasmids or with an empty vector control by using Lipofectamine 2000 (Invitrogen Inc). Forty eight hour after transfection, cells were selected in G418 (500 μg/ml) containing medium for 3 weeks. Several independent colonies were isolated, expanded, and analyzed for protein expression. For transient co-transfection assay, HEK293 cells were transfected with 3 μg each HA-XIAP and p3X-CMV-FLAG Casp3 WT, Casp3-Zn-mutants or empty vector (p3X-CMV-FLAG plus pcDNA3.1) by using FuGENE6 (Promega Inc) transfection reagent. Forty eight hour after transfection cells were harvested for immunoprecipitation analysis.

### Immunoprecipitation and co-IP

Sub-confluent stably over-expressed DKO MEF cells (in 100-mm plates) were lysed in IP buffer (Pierce) and an equal amount of lysates (3000 μg) was immunoprecipitated overnight with EZview red anti-Flag (25 μl/mg protein) affinity gel. For co-IP assay, HEK-293 cells were transfected with 3 μg each of HA-XIAP and p3XCMV-FLAG-Casp3 WT or Casp3-Zn mutant plasmids. At 48 hr post-transfection, cells were lysed in IP buffer and an equal amount of lysates (500 μg) was immunoprecipitated overnight with EZview red anti-Flag or EZview red anti-HA affinity gel (Sigma). Following immunoprecipitation, the beads were washed 3 times with lysis buffer, resuspended in 2X Laemmli sample buffer (Bio-Rad Lab), fractionated on SDS-12% polyacrylamide gel and analyzed by Western blot with rabbit anti-HA (Bethyl Lab, TX, USA) or with mouse M2-FLAG (Sigma) antibodies.

### Western blot analysis

Protein lysates and immunoblots were prepared as described [18]. Protein band quantification was done using a LI-COR Odyssey CLx Infrared Imaging System.

### Total RNA extraction and real time PCR assay

Total RNA from MEF cells was prepared [17] by using RNeasy Mini Kit (Qiagen, 74106) and quantitated using NanoDrop ND 1000 spectrophotometer (Thermo Fisher Scientific). Real time PCR was performed using 7900HT Fast Real-Time PCR detection system (Applied Biosystems). Primers and probes used for mRNA detection were as follow: CASP3 (Hs00234387_m1), XIAP (Mm01311594_mH), and GAPDH (Hs03929097_g1).

### Flow cytometry

Briefly 10^6^ cells were stained with Annexin V FITC followed by propidium iodide staining and acquired on a FACS Calibur (BD Biosciences) and analyzed using Flow Jo software (TreeStar) as previously described [17].

### Immunofluorescence

Jeko-1 cells were cyto-spinned on slides [18] following B-PAC-1 treatment. Cells were fixed in 2% paraformaldehyde, permeablized with 0.2% Triton X-100, and stained with cleaved anti-rabbit Casp3 antibody and with 1:100 diluted Hoechst stock solution in H_2_O using standard protocol. Cells were mounted with ProLong Gold Antifade (Life Technologies Inc) and scanned in a Labophot-2 fluorescence microscope (Nikon, Tokyo, Japan).

### Statistical analysis

Statistical analysis was done using Prism (GraphPad Software, San Diego, CA). All numerical results are presented as means ± SEM. The statistical significance of differences was analyzed using Student's *t*-test (paired) or by Anova analysis.

## SUPPLEMENTARY FIGURES AND TABLES


