# Supplementary material for: Molecular evidence of Zn chelation of the procaspase activating compound B-PAC-1 in B cell lymphoma
Source: Oncotarget. 2015 Dec 7;7(3):3461–76. doi: 10.18632/oncotarget.6505 (PMC4823120; doi:10.18632/oncotarget.6505)
Supplement: Supplementary file 1 [file oncotarget-07-3461-s001.pdf]

SUPPLEMENTARY FIGURES AND TABLES

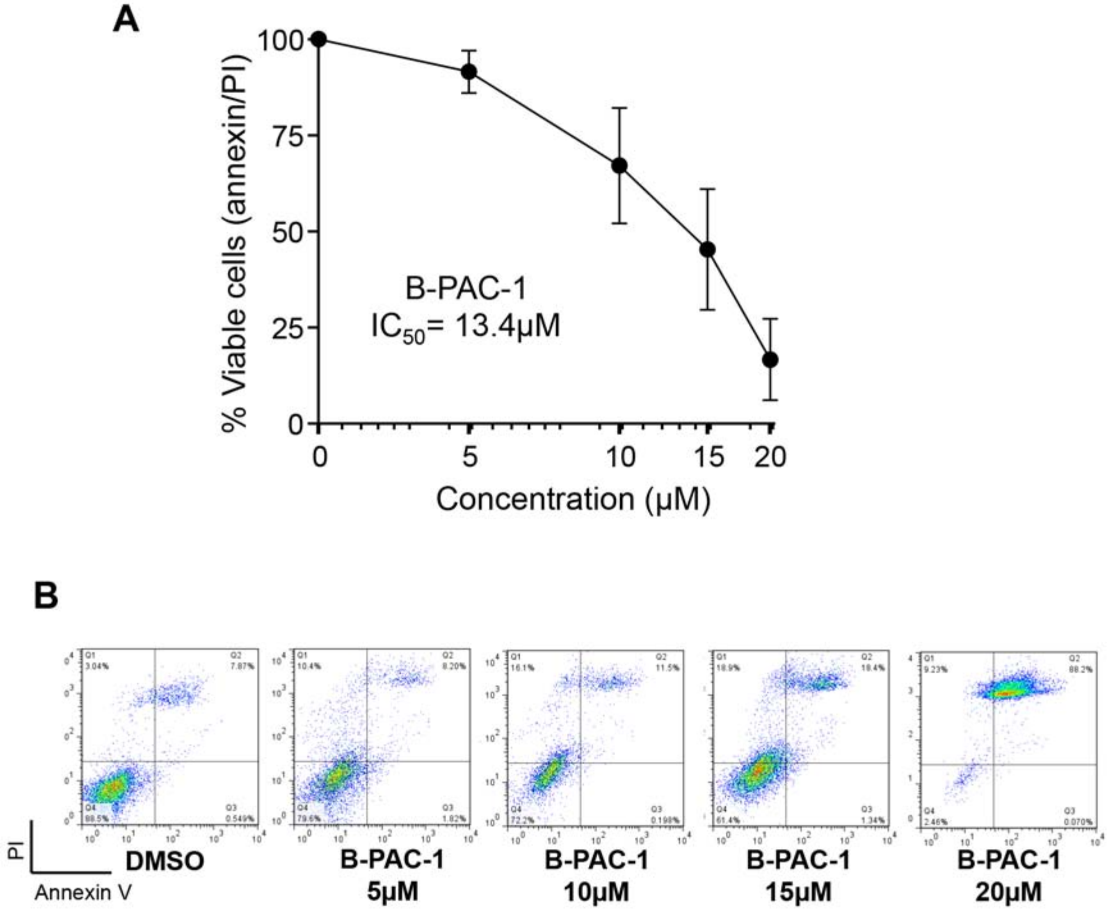

Supplementary Figure S1: A. Dose dependent cell death by B-PAC-1 in MEF cells stably over-expressed with WT Casp3 construct. 0.5 × 10^6 cells were treated with 5, 10, 15 or 20 μM B-PAC-1 for 24 hr. Cell were harvested and stained with Annexin V and PI and the amount of apoptosis was measured compared with DMSO control. Mean ~ SE (*n* = 7); IC_{50} value was determined as shown in the inset (13.4 μM). **B.** Representative Annexin V-PI FACS analysis showing dose dependent induction of apoptosis induced by B-PAC-1

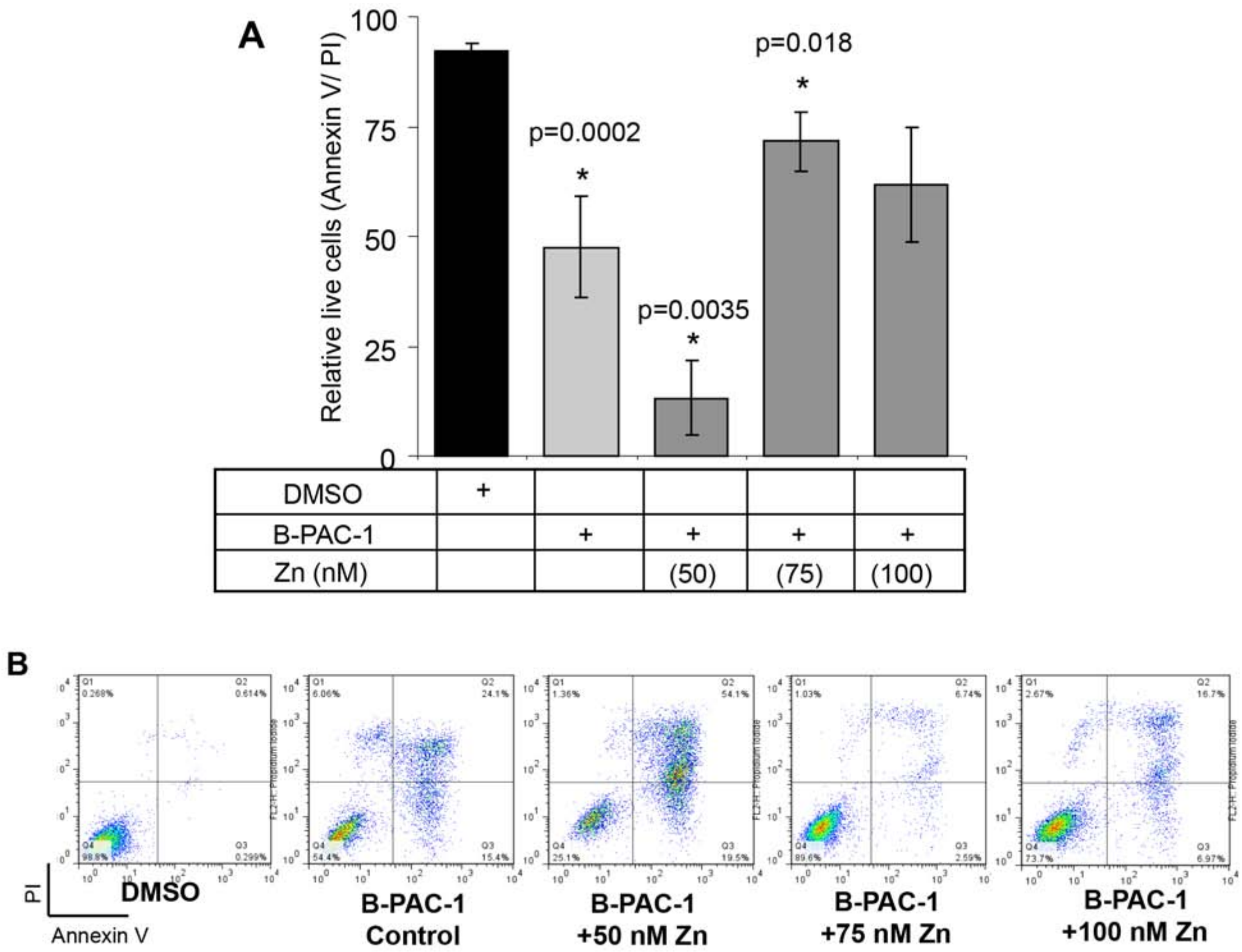

Supplementary Figure S2: A. Dose dependent inhibition of B-PAC-1 induced apoptosis of WT Casp3 expressing MEF cells by exogenous Zn. Stably over-expressed WT Casp3 (0.5×10^6/well) MEF cells were treated with 15 µM B-PAC-1 either alone or in combination with Zn in the indicated amount for 24 hr. Cell were harvested and stained with Annexin V and PI and the amount of apoptosis was measured. Mean ± SE ($n = 5$); *significant difference from DMSO treated cells. Maximum protection was calculated as 90 nM of Zn. **B.** Representative Annexin V-PI FACS analysis showing dose dependent Zn inhibition of apoptosis induced by B-PAC-1.

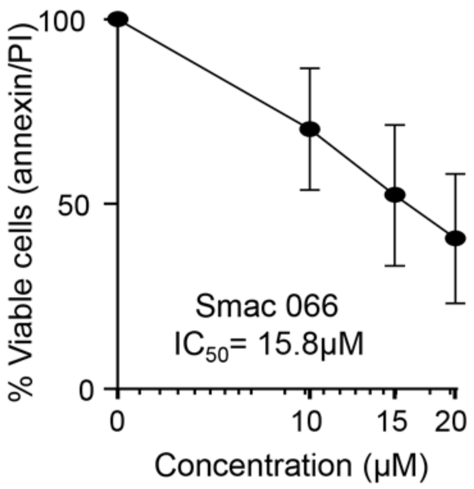

Supplementary Figure S3: Dose dependent cell death by 066 in MEF cells stably over-expressed with WT Casp3. 0.5 × 10^6 cells were treated with 10, 15 or 20 µM 066 for 24 hr. Cell were harvested and stained with Annexin V and PI and the amount of apoptosis was measured compared with DMSO control. Mean ± SE (n = 5); IC_{50} value was determined as shown in the inset (15.8 µM)

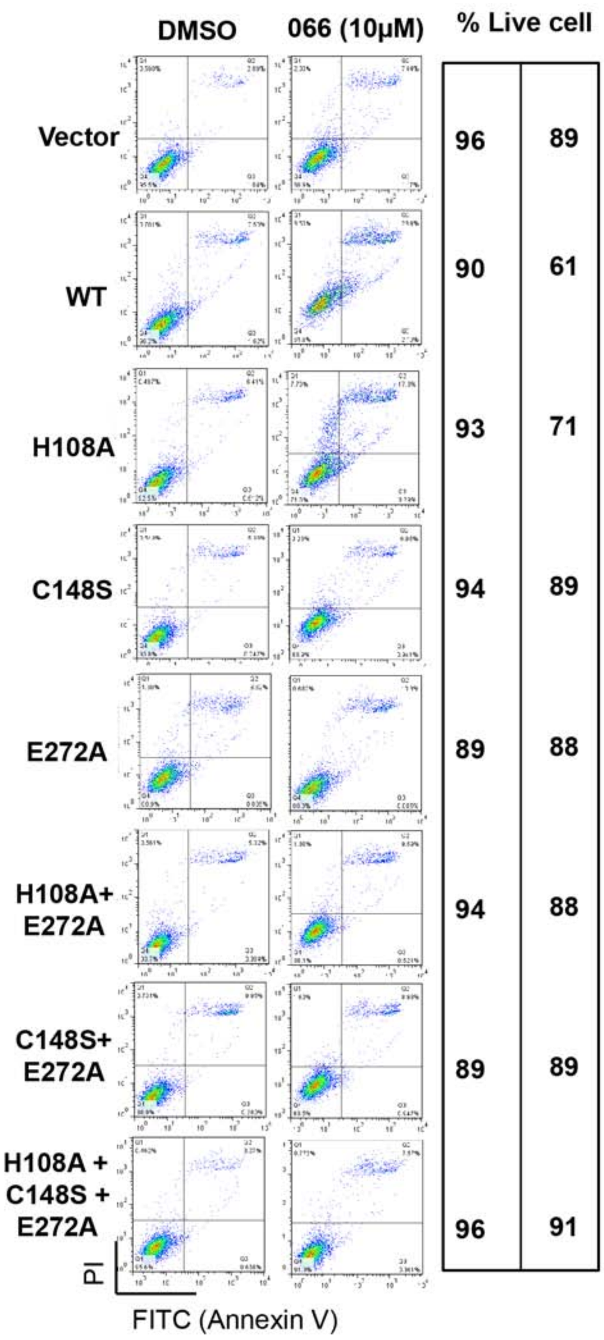

Supplementary Figure S4: Representative Annexin V-PI FACS analysis of smac mimetic 066 induced cell death. MEF cells stably over-expressed with indicated plasmids were seeded into 6 well plate (0.5 ± 10^6/well) and treated with 10 μM 066 for 24 hr. Cells were harvested and stained with Annexin V and PI and the amount of apoptosis was measured

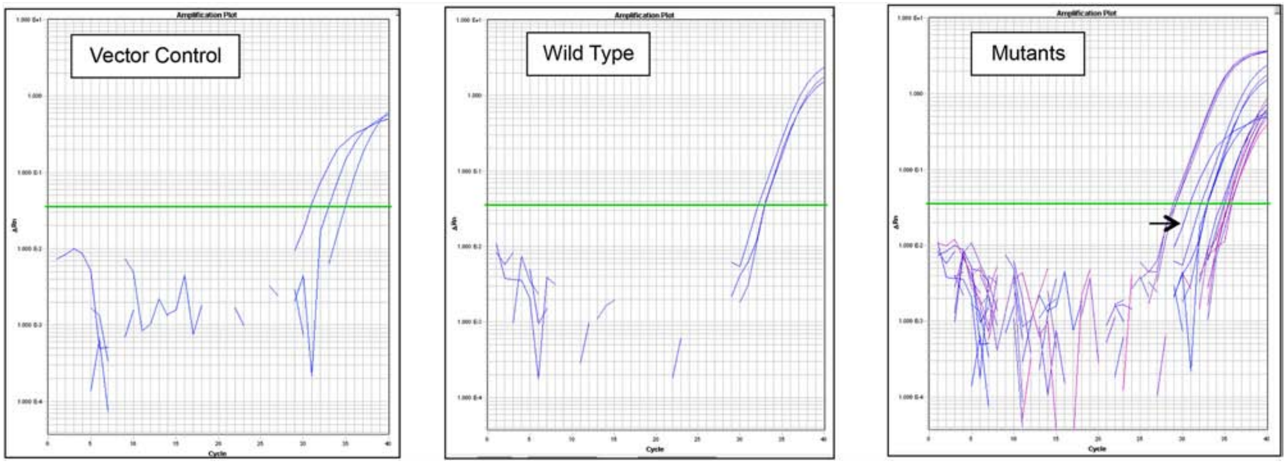

Supplementary Figure S5: qRT-PCR expression of Casp3 in vector control, WT and Zn-Casp3 mutant MEFs. Representative snapshot of results of SDS 2.3 software showing amplification curve. Black arrow showed the amplification curves of vector control compared to other MEFs

Supplementary Table S1: List of antibodies and reagents used.

Antibodies/reagents	Source and Catalog #
Casp3	Cell Signaling, 9665
Casp9	Cell Signaling, 9502
Casp6	Cell Signaling, 9762
Casp7	Cell Signaling, 9492
cIAP2	Epitomics, S2700
cIAP1	Abcam, ab2399
Mcl-1	Santa Cruz, S-19
ATM	EMD Millipore, PC116
Smac (DIABLO)	BD Biosciences, 612246
XIAP	BD Biosciences, 610762
Poly (ADP-ribose) polymerase	Enzo Life Sciences, BML-SA250
Cleaved Poly (ADP-ribose) polymerase	Cell Signaling, 9541
Rabbit HA	Bethyl Lab., A190-108P
Mouse FLAG	Sigma-Aldrich, F1804
GAPDH	Sigma-Aldrich, G8795
Staurosporine	LC Laboratories
Bendamustine	Selleckchem
MK2206	Selleckchem
ABT199	Xcessbio, CA
Annexin V fluorescein isothiocyanate (FITC),	BD Biosciences
Hoechst 33258	Invitrogen
Zinc sulfate heptahydrate (Zn. $7H_20$)	Sigma-Aldrich
Geneticin® (G418 Sulfate) (50 mg/mL)	Life Technologies (10131–035)
FuGENE6	Promega Inc
cycloheximide	Sigma-Aldrich
